# Effectiveness of negative-pressure wound therapy in partial-thickness and full-thickness burns: a systematic review

**DOI:** 10.1590/acb415426

**Published:** 2026-09-11

**Authors:** Vanessa Cindy Oliveira Neres Lima de Souza, Ana Caroliny da Silva, Ruy de Souza Lino, Thauana Garcia da Silveira

**Affiliations:** 1 Universidade Federal de Goiás – School of Nursing – Goiânia (GO) – Brazil.; 2 Universidade Federal de Goiás – Tropical Pathology and Public Health Institute – Department of Biosciences and Technology – Goiânia (GO) – Brazil.

**Keywords:** Wound Healing, Burns, Negative-Pressure Wound Therapy

## Abstract

**Purpose:**

To evaluate the effectiveness of negative-pressure wound therapy (NPWT) in healing partial- and full-thickness burns in humans.

**Methods:**

This systematic review followed the Cochrane Handbook for Systematic Reviews of Interventions and the PRISMA checklist.

**Results:**

Eight studies published between 2014 and 2024 were included, involving 466 patients with partial- and full-thickness burns. The sample comprised five randomized controlled trials, two retrospective observational studies, and one prospective cohort study. NPWT was applied in continuous or intermittent modes, with pressures ranging from 40 to 125 mmHg. The most frequently assessed outcome was re-epithelialization time, with NPWT superiority reported in three studies. NPWT was also associated with greater reduction in wound area and bacterial load. Additional outcomes included pain, graft integration, and scar quality, as well as logistical advantages such as reduced dressing change frequency.

**Conclusion:**

NPWT provides benefits in burn treatment, including reduction of wound area, bacterial count, and re-epithelialization time, along with effectiveness in graft integration and healing quality. Despite limitations, such as application difficulties and prolonged treatment in pediatric patients, NPWT stands out for decreasing dressing changes, improving care logistics and psychological well-being.

## Introduction

As the primary interface with the external environment, the skin is subject to various forms of trauma over the lifespan, including burns, defined as damage to the skin or any organic tissue, caused mainly by thermal, chemical, electrical, radioactive agents, or friction. Burns constitute a heterogeneous group of injuries that can be classified according to the depth of tissue damage and are categorized as superficial thickness, partial thickness (superficial or deep), and full thickness^1-3^.

Unlike partial-thickness burns, which preserve remnants of the dermal appendages and have the potential for spontaneous re-epithelialization, full-thickness burns result in complete destruction of the epidermis and dermis, compromising tissue regeneration, and therefore, require surgical interventions such as skin grafting^4^.

From an epidemiological perspective, burns are a significant global public health problem. The World Health Organization (WHO) estimates that approximately 180,000 deaths occur annually because of these injuries, with the majority concentrated in low- and middle-income countries, where access to specialized services and advanced care technologies is limited^5,6^.

Burn treatment does not always provide an ideal environment for tissue regeneration, especially in deep burns or those with high exudate production. This has driven the incorporation of adjuvant therapies, such as negative-pressure wound therapy (NPWT), in the management of these injuries^7^.

NPWT was introduced commercially in 1997 and consists of a system of three essential components: an open-cell foam, a semi-occlusive dressing, and a controlled subatmospheric pressure source. The application of this system promotes exudate removal, edema reduction, increased local perfusion, wound margin contraction, and the formation of a microenvironment favorable to cell proliferation and angiogenesis^8-10^.

Several studies suggest benefits of NPWT in the treatment of burns of varying depths, including reduced bacterial load, accelerated re-epithelialization, and improved skin graft integration^11-13^. However, limitations related to variability in application protocols and methodological heterogeneity between studies persist, making it difficult to draw consistent conclusions about their actual magnitude of effectiveness.

NPWT in burn care still has significant limitations, such as high cost, the need for specific equipment, risk of bleeding, and heterogeneity of results depending on the depth and extent of the injury among the available studies. Given this scenario, there is a clear need for a critical and up-to-date summary of the available evidence on the use of NPWT in the treatment of burns. Therefore, the aim of this study was to evaluate the effectiveness of NPWT in the healing of partial- and full-thickness burns in humans.

## Methods

This is a systematic review developed to evaluate the effect of NPWT in the treatment of partial- and full-thickness burns. The review followed the recommendations of the Cochrane Handbook for Systematic Reviews of Interventions^14^. The study report was structured according to the checklist of the Preferred Reporting Items for Systematic Reviews and Meta-Analyses (PRISMA) 2020^15^. The full PRISMA 2020 checklist is provided as Suppl. Mat. 1^16^. The review protocol was previously registered at International Prospective Register of Systematic Reviews (PROSPERO), under registration number CRD42024576732.

The guiding question of the review was formulated using the PICO (Population, Intervention, Comparison, Outcomes) strategy, as recommended for systematic reviews of interventions^15^, and was as follows: What is the effect of NPWT on the healing process of partial- and full-thickness burns?

- P (Population): individuals of any age with partial- or full-thickness burns;
- I (Intervention): NPWT;
- C (Comparison): conventional treatment or other approaches described in the included studies, according to the methodological design of each study;
- O (Outcomes): parameters related to healing, including re-epithelialization time, granulation tissue formation, graft integration rate, infection control, pain intensity, hospital stay, and other relevant clinical variables.

The electronic search was performed in the following databases: MEDLINE (PubMed), Web of Science, Embase, Latin American and Caribbean Health Sciences Literature (LILACS), Virtual Health Library, and Scopus, using the advanced search mode. Studies published between June 2014 and July 2025 were included, without language restrictions. The inclusion period (2014–2025) was defined to capture more recent evidence consistent with current clinical practices in burn management.

To develop the search strategy, Medical Subject Headings (MeSH) terms were selected using a conceptual mapping approach^16^, as described by Ribeiro-Rotta et al.^17^. The search strategies were subsequently adapted to the specific characteristics of each database^16^ .

The inclusion criteria were randomized controlled trials and observational studies that evaluated NPWT as the primary intervention or as a central adjunctive treatment, including its use for wound bed preparation or skin graft fixation, in partial- and full-thickness burns in humans.

The exclusion criteria were review studies, experimental animal studies, case reports or case series, letters to the editor, recommendations and guidelines, and studies in which NPWT was used only as a secondary or supportive component of combination therapies, without isolated evaluation of its clinical effects.

The screening and organization of articles were conducted using Rayyan software, which was used to identify duplicates, organize references, and optimize the selection process^18^. Two reviewers independently (TGS and VCONLS) read the titles and abstracts, following the previously established eligibility criteria. Studies considered potentially eligible were read in full. Disagreements between reviewers were resolved by a third evaluator (RSLJ). Reasons for exclusion after full-text review are presented in Suppl. Mat. 4^16^.

**Figure 1:**
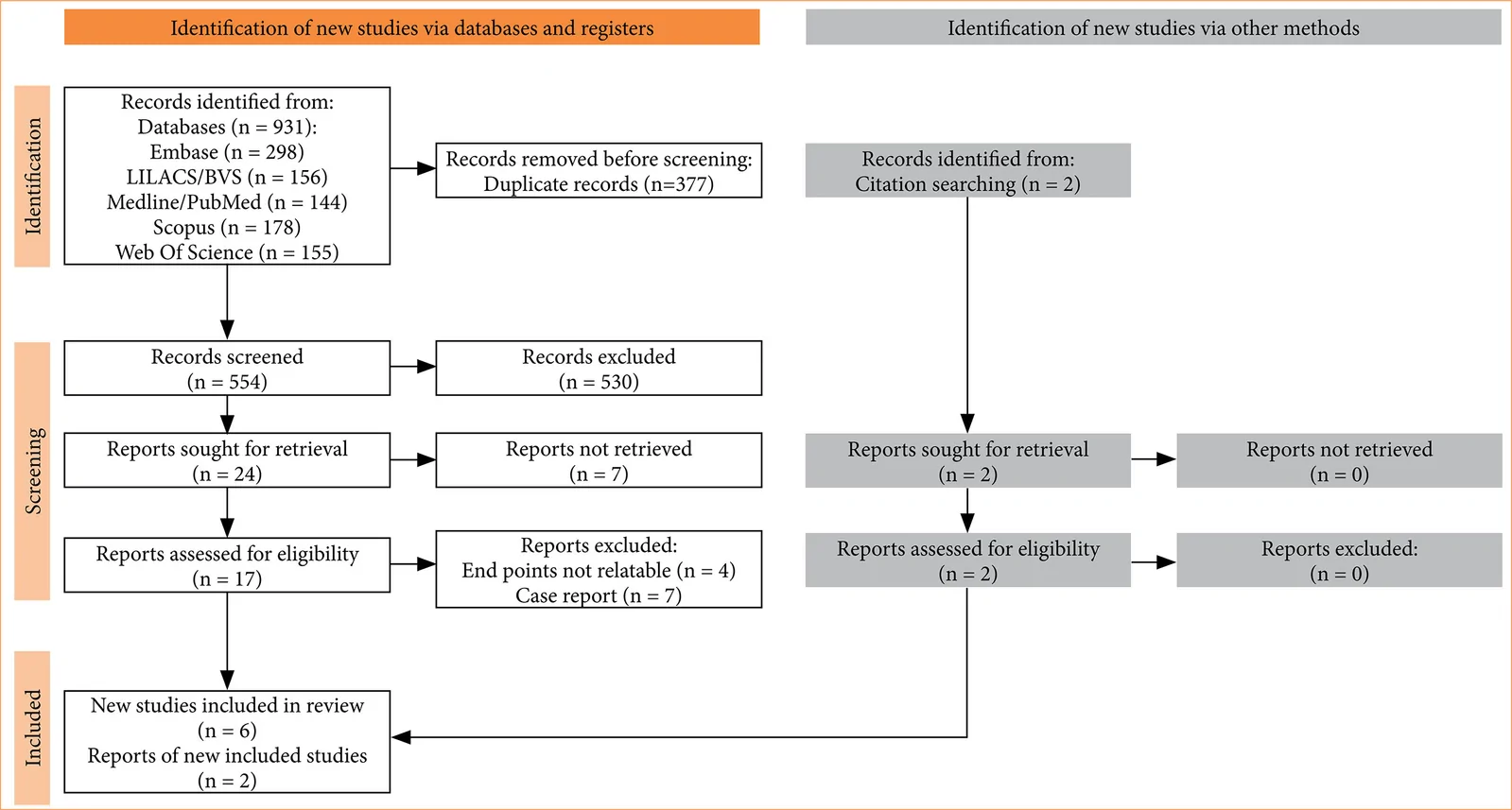
Preferred Reporting Items for Systematic Reviews and Meta-Analyses (PRISMA) 2020 flowchart.

LILACS: Latin American and Caribbean Health Sciences Literature; BVS: Virtual Health Library. Source: Haddaway et al.^29^.

The selection process was documented using a flowchart in accordance with PRISMA guidelines (Fig. 1). Data from the included studies were extracted and recorded in a Microsoft Excel spreadsheet. The following variables were collected: title, DOI, study type, country, year of publication, study design, burn classification, study population, sample size, affected body surface area, intervention, comparison groups, therapy-related complications, outcomes, treatment duration, and statistical findings.

The risk of bias assessment was conducted by two reviewers independently (TGS and VCONLS). For randomized controlled trials, the Cochrane Risk of Bias 2.0 (RoB 2) tool was used^19^. For non-randomized observational studies, Risk of Bias in Non-randomized Studies of Interventions (ROBINS-I) was used^20^.

## Results

Eight studies were included in this systematic review, of which two retrospective observational studies^21,22^, five randomized controlled trials^23-27^, and one prospective cohort study^28^, including a total of 466 patients with total body surface area (TBSA) burned ranging from 0.5 to 40%.

The publications included data from the period between 2014 and 2024 and involved individuals with partial- and full-thickness burns. The process of identification, screening, eligibility, and inclusion of studies is described in the PRISMA flowchart, shown in Fig. 1. The data extracted from these eight studies formed the basis of this review and are organized in Table 1.

**Table 1:** Characteristics and main outcomes of studies evaluating negative-pressure wound therapy in burn injuries.

Authors	Study design	Population (n; age)	Burn depth	NPWT protocol	Comparator	Primary outcomes	Main findings
Waltzman and Bell^21^	Retrospective observational	67 patients;1–84	Full thickness	Continuous NPWT, -125 mmHg, post-grafting	None (single arm)	Re-epithelialization time, need for graft repetition, grafted area, percentage of graft	No graft failure occurred, and the mean re-epithelialization time was 16 ± 7 days
Ehrl et al.^22^	Retrospective observational	30 patients (41 hands); adults	Partial- and full-thickness (hand burns)	Continuous NPWT, -75 mmHg for 5–7 days	None (single arm)	Scar, function of upper and lower limbs	Good scar quality in most cases, and low incidence of hypertrophic scarring
Ibrahim et al.^23^	Randomized controlled trial	45 patients; 20–40	Partial thickness	Intermittent NPWT, -125 mmHg	MES and standard care	Bacterial load, wound closure rate	NPWT was superior to the control group in reducing wound area and bacterial load at 21 days
Frear et al.^24^	Randomized controlled trial	101 children; 1–9	Partial thickness	Continuous NPWT, -80 mmHg	Silver mesh dressing	Re-epithelialization, pain, scar, grafted area, pruritus, wound perfusion, adverse events, and ease management	No significant clinical superiority was observed, but NPWT was associated with greater application complexity
Sun et al.^25^	Randomized controlled trial	118 patients; adults	Full thickness	Continuous NPWT with biotranslucent membrane	Iodine-soaked gauze	Re-epithelialization time, pain, bacterial load, number of days of treatment up to the bed is ready for grafting, survival rate, use of antibiotics, hospital stay length	NPWT improved graft survival and reduced infection rates, pain, antibiotic use, and hospital stay length
Tapking et al.^26^	Randomized controlled trial	61 patients; ≥ 18	Partial thickness	NPWT, -80 mmHg (mode not reported)	Conventional dressings	Re-epithelialization time, pain, scar, function of upper and lower limbs, number of dressing changes, quality of life	NPWT significantly reduced the number of dressing changes and provided logistical and psychological benefits
Hsiao et al.^27^	Randomized controlled trial	28 patients; 24–76	Partial thickness	Occlusive NPWT drainage system	Moist dressing + splint	Pain, need for graft repetition, scar, wound closure rate, pruritus, clinical infection	Similar graft take was observed in both groups, but NPWT was associated with lower pain levels and higher patient satisfaction
Babu et al.^28^	Prospective cohort	16 patients; 18–72	Partial- and full-thickness	Continuous NPWT, -70 to -100 mmHg	Conventional graft dressing	Re-epithelialization time, bacterial load, need for graft repetition, percentage of graft, number of dressing changes	NPWT reduced graft loss and the duration of dressing use, while increasing the re-epithelialization rate

NPWT: negative-pressure wound therapy; MES: microcurrent electrical stimulation. Source: Elaborated by the authors.

**Figure 2:**
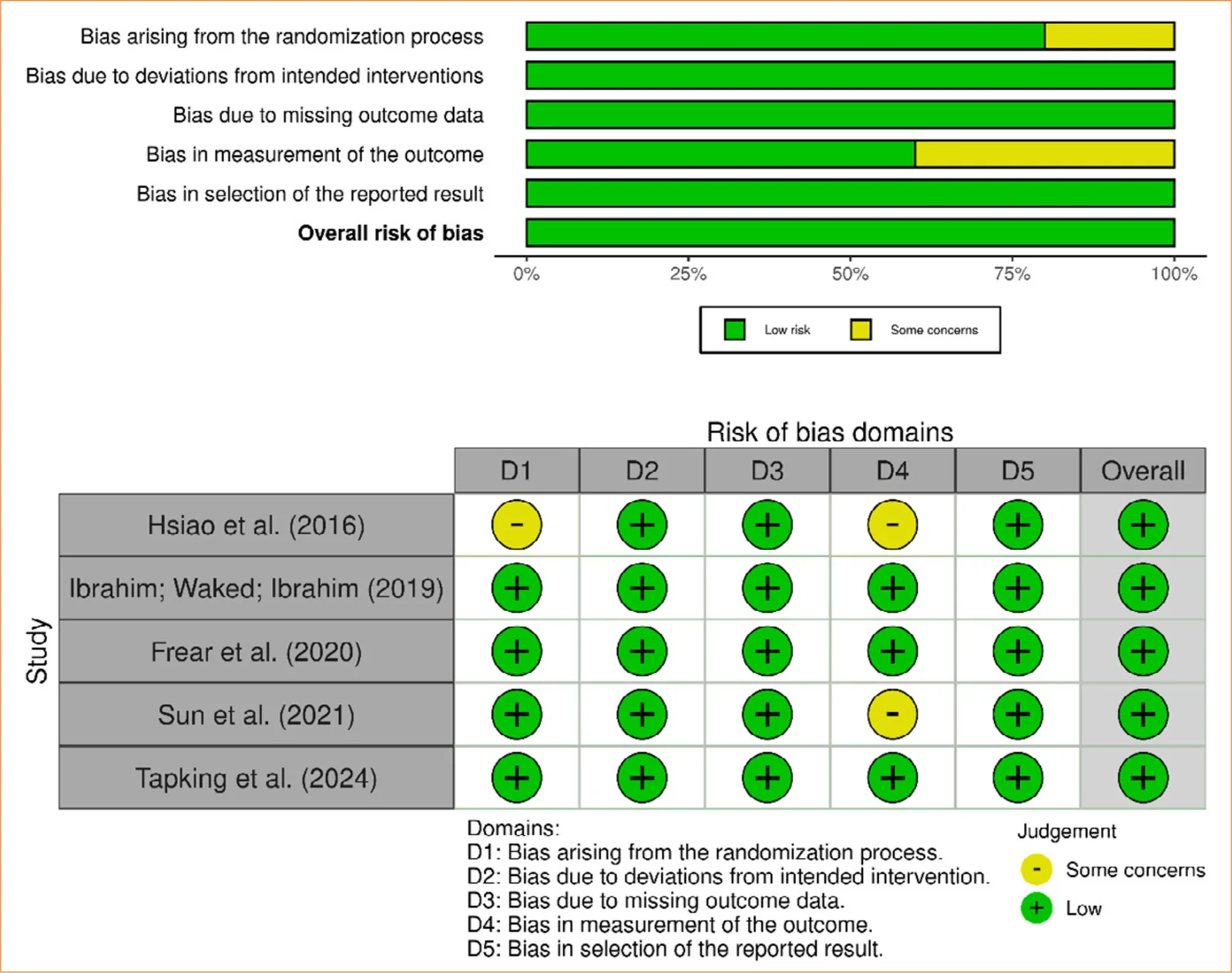
Risk of bias graph of randomized controlled trials (RoB 2.0).

Source: Elaborated by the authors.

The risk of bias of randomized controlled trials was assessed using the Cochrane Risk of Bias 2.0 (RoB 2) tool. Overall, most studies were judged to present low risk of bias across the main evaluated domains, as illustrated in Fig. 2. However, differences in study design and relatively small sample sizes were observed across trials, which should be considered when interpreting the findings. These factors may limit the precision of effect estimates, despite the overall favorable methodological assessment.

**Figure 3:**
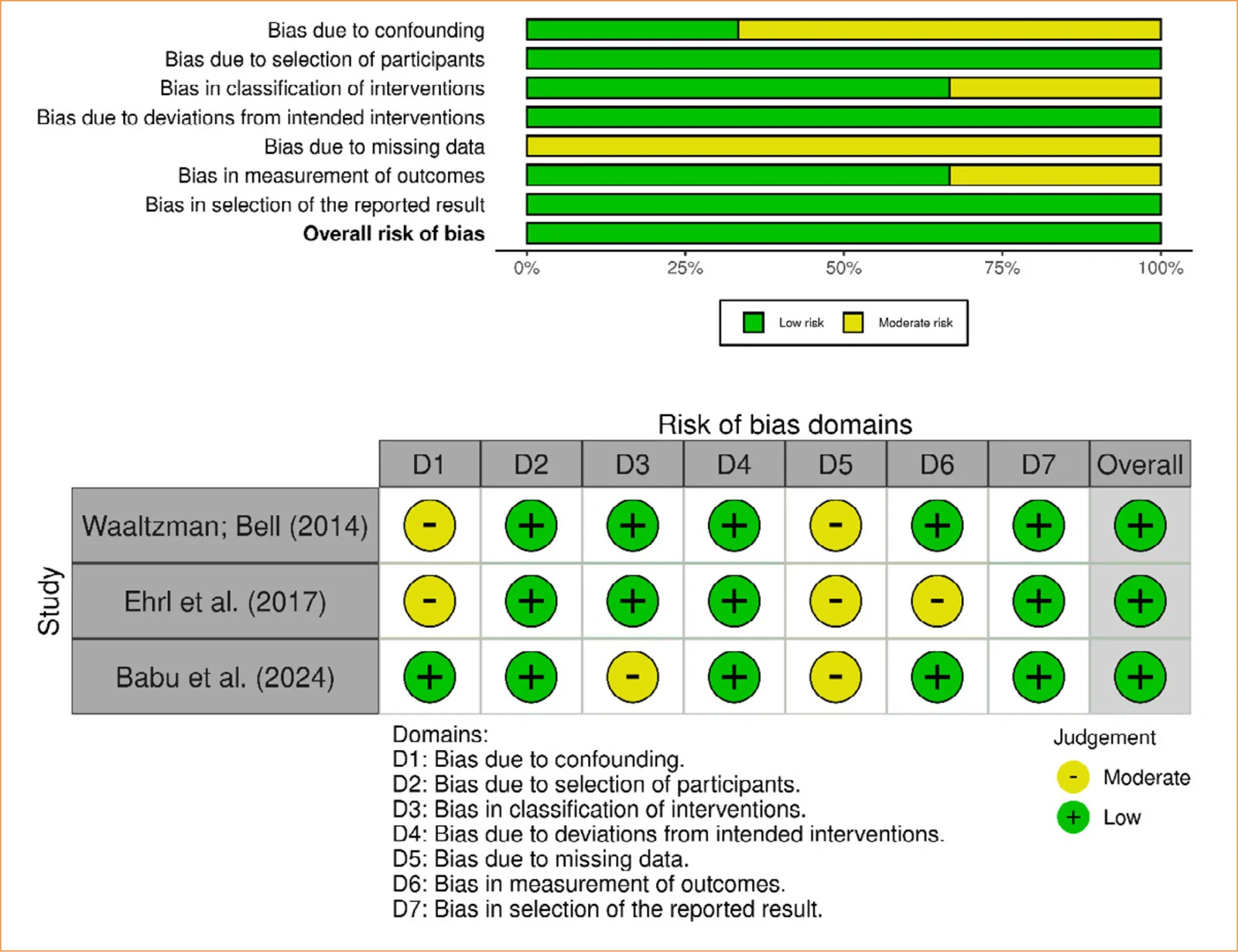
Risk of bias graph of non-randomized controlled trials (ROBINS-I).

Source: Elaborated by the authors.

The risk of bias in retrospective observational studies and the prospective single-center cohort was evaluated using the ROBINS-I tool. Although some methodological limitations were identified in specific domains, particularly those inherent to observational designs, the included studies demonstrated acceptable overall methodological quality. Consequently, the risk of bias was considered low to moderate, as presented in Fig. 3.

Among the different modes of NPWT application, six studies used the continuous mode^21,^ ^22,^ ^24,^ ^25,^ ^27,^ ^28^, and one study used the intermittent mode^23^. The pressures applied ranged from 40 to 125 mmHg, with 125 mmHg being the most common. Different treatments were used in the control groups of the randomized studies. In the study by Tapking et al.^26^, patients treated with debridement and dressings containing polyhexanide, oily gauze, and cotton changed every 48 hours, compared with patients who underwent tangential surgical debridement, and received skin grafts followed by additional NPWT. The study by Ibrahim et al.^23^ evaluated the effectiveness of microcurrent electrical stimulation (MES) and standard wound care compared to NPWT, highlighting technological approaches to stimulate healing. Frear et al.^24^ used a silver mesh as a standard dressing in the control group. Sun et al.^25^ used iodine-soaked gauze as a dressing. In all groups, initial debridement was performed, in addition to cleaning the wound at each dressing change.

The primary outcomes assessed across the selected studies included re-epithelialization time, graft integration, bacterial load, pain, and scar quality. The most frequently investigated outcome was re-epithelialization time, assessed in five of the eight studies^21,24-26,28^. Among these, Sun et al.^25^ and Babu et al.^28^ showed statistically significant differences in favor of NPWT. Sun et al.^25^ demonstrated a significantly shorter wound closure time, approximately five days less than in the control group (*p* < 0.05). In the study by Babu et al.28, the percentage of re-epithelialization was greater than 90% in a significantly higher proportion of patients in the NPWT group (*p* = 0.036). In the study by Frear et al.^24^, NPWT significantly reduced time to re-epithelialization compared with the control group, based on adjusted models accounting for burn depth, anatomical location, and etiology incidence rate ratio (IRR) = 0.78, 95% confidence interval [95%CI] 0.66–0.93; *p* = 0.005). Ibrahim et al.^23^, although did not measure complete re-epithelialization, demonstrated a significantly greater reduction in wound area in the group undergoing NPWT after 21 days of treatment compared with the standard care control group (*p* < 0.001).

The reduction in bacterial load also showed a statistical difference in favor of NPWT, especially in the study by Ibrahim et al.^23^, which reported, on day 10, a lower mean bacterial count (*p* < 0.001) in the NPWT group, whereas the control group showed the highest mean bacterial count (84.1 ± 17.0) (*p* < 0.001).

## Discussion

Since its introduction into clinical practice in 1997, NPWT has been increasingly adopted in the management of complex wounds, including burn injuries^9,30^. In this context, the present systematic review aimed to synthesize the available evidence regarding the effectiveness of NPWT in the treatment of partial- and full-thickness burns. Overall, the findings indicate that NPWT is associated with favorable clinical outcomes when compared with conventional dressings, including a reduction in wound area, decreased bacterial burden, accelerated re-epithelialization, and improved skin graft integration in selected scenarios^31^. These results support the role of NPWT as an adjunctive therapeutic strategy across different stages of burn care, particularly in complex wounds requiring optimized wound bed preparation and graft stabilization. However, despite the consistency of positive outcomes reported in the literature, the substantial clinical and methodological heterogeneity among the included studies highlights the need for cautious interpretation and reinforces the importance of individualized treatment protocols. In addition, part of the available evidence is derived from observational studies without control groups, which does not allow for direct inference regarding comparative effectiveness.

The application of NPWT in different degrees of burn severity, including partial- and full-thickness injuries, has demonstrated favorable outcomes in multiple anatomical regions, with evidence of efficacy in body areas ranging from 0.5 to 10% of the TBSA. Studies also point to significant benefits in patients with more extensive burns (≥ 15% TBSA), including reduced risk of infection, improved re-epithelialization, and greater efficiency in fluid control, which are crucial aspects for clinical stabilization and favorable clinical outcomes in these patients^32^.

The results of this review indicate that NPWT not only promotes wound closure but also has a positive impact on functional and reconstructive outcomes, particularly in the context of skin graft integration. The incorporation of NPWT in the acute phases of treatment was associated with graft integration rates close to 100%, underscoring its role as a key adjunct for the adequate preparation of the wound bed and for the success of the surgical procedure. NPWT enhances graft integration by stabilizing the graft–wound interface, minimizing shear forces, and reducing hematoma and seroma formation, thereby facilitating capillary inosculation and graft adherence. These findings are further supported by a meta-analysis demonstrating that NPWT significantly increases graft integration rates and reduces infection rates when compared with conventional therapies, reinforcing its superiority in certain clinical scenarios^11^.

In addition to conventional dressings, several other strategies have been employed for burn wound bed preparation, including dermal substitutes and skin allografts. Dermal substitutes such as Integra, Matriderm, and NovoSorb BTM have emerged as important tools in the management of deep partial- and full-thickness burns, particularly in patients with limited donor sites or exposed tendon and bone, due to their ability to promote neodermis formation and improve long-term scar quality^33,34^. However, these approaches may require staged surgical procedures and, in some settings, have been associated with delayed re-epithelialization and higher treatment costs^33^. Skin allografts remain an important temporary coverage strategy in extensive burns, helping reduce fluid loss, pain, and infection risk while preparing the wound bed for definitive autografting^35^. Rather than acting as a skin substitute itself, NPWT optimizes the wound environment through edema reduction, exudate control, improved perfusion, and graft stabilization. Therefore, NPWT may play a complementary role alongside dermal substitutes and allografts, particularly in complex wounds requiring enhanced wound bed preparation and graft integration.

In burns affecting functionally critical areas, such as the hands, NPWT appears to offer additional benefits in terms of preserving mobility and scar retraction, and improving aesthetic and functional appearance. Patients with partial- and full-thickness burns on their hands treated with NPWT showed significant long-term maintenance of finger mobility and better-quality scar formation^22^. Similar results were observed in a study that associated NPWT with an early rehabilitation protocol in patients with partial-thickness burns on the hands, whose areas ranged from 0.5 to 2% of TBSA, showing functional improvement and acceleration of the healing process^36^. These findings reinforce the importance of NPWT as a resource integrated into multidisciplinary rehabilitation strategies.

Another relevant aspect concerns the definition of the ideal subatmospheric pressure to be used during treatment. In the studies included in this review, the pressures applied predominantly ranged between -75 and -125 mmHg^21–23,26^, except for pediatric patients younger than 12 months old, in whom a reduced pressure of 40 mmHg was used^24^. These parameters are in line with the recommendations of the European Wound Management Association, which establishes the range between -75 and -125 mmHg as the most widely indicated for stimulating granulation tissue formation, wound contraction, and exudate removal^9^. According to this document, pressures close to -125 mmHg are traditionally used to maximize angiogenesis and granulation tissue formation, while moderate pressures (-75 to -100 mmHg) are preferable in more sensitive or compromised tissues, due to the lower risk of hypoperfusion and local ischemia, in addition to the ability to modulate cytokines and stimulate growth factors, such as vascular endothelial growth factor and basic fibroblast growth factor. Excessive high pressures can induce pain and potential tissue damage, while very low pressures tend to be ineffective, highlighting the need for individualized therapy according to the characteristics of the wound and the patient^9^.

This need for dynamic pressure adjustment is evidenced in reports such as the one by Gómez-Ortega et al.^37^, who described the management of patients with electrical burns using, initially, a pressure of -125 mmHg for five days and, after grafting, a reduction to -100 mmHg for another four or five days. This strategy resulted in graft integration rates ranging from 85 to 100%, emphasizing the usefulness of NPWT both in the preparatory phase for reconstruction and in post-grafting support, especially in deep lesions with a high risk of complications^38^.

The most frequently evaluated outcome in this review was the time to re-epithelialization, which is recognized as one of the main indicators of therapeutic efficacy in wounds. Re-epithelialization is directly related not only to the restoration of skin integrity but also to functional recovery and reduced risk of secondary infections and may be enhanced by NPWT through the maintenance of a moist wound environment, reduction of interstitial edema, and improvement of local perfusion, which collectively facilitate keratinocyte migration and proliferation^21,24-26^.

Although the study by Waltzman and Bell^21^ found no statistically significant association between the occurrence of burns and the variables of age and gender, the literature consistently recognizes the impact of social determinants on the distribution of these events. Epidemiological evidence indicates that children and adult women are among the groups most vulnerable to burns, which may be related to factors such as greater exposure to heat sources at home, socioeconomic inequalities, and unsafe working conditions^39^.

In addition, the reduction in microbial load associated with NPWT use represents one of its most relevant mechanisms in the context of burns, since secondary infections remain one of the leading causes of morbidity and mortality in these patients. The study by Ibrahim et al.^23^ demonstrated a statistically significant reduction in microbial colonization in wounds treated with NPWT, a finding of extreme relevance given the risk of progression to sepsis and septic shock, a complication known to be associated with high mortality rates in patients with extensive burns. Continuous exudate removal, decreased tissue edema, improved oxygenation, and disruption of bacterial biofilms create a less favorable environment for microbial proliferation^40^.

NPWT has demonstrated a favorable cost–benefit profile, particularly when both clinical and economic outcomes are considered. In a randomized controlled trial involving pediatric burn patients, NPWT was associated with a mean cost per patient of AUD $903.69 compared to AUD $1,669.01 for standard care, resulting in an average cost savings of $948.57 per patient, in addition to a reduction in time to re-epithelialization and a high probability (89%) of being a dominant strategy^41^. Although these values are reported in Australian dollars, they reflect a substantial reduction in overall healthcare costs. Furthermore, a systematic review and meta-analysis found that NPWT was associated with a higher likelihood of wound healing (*odds ratio* = 1.56; 95%CI 1.15–2.13) and a reduction in hospital length of stay by an average of 4.78 days, which indirectly contributes to cost savings, although no direct monetary estimates in U.S. dollars were reported^42^. Therefore, despite potentially higher initial costs related to devices and materials, NPWT tends to be cost-effective by reducing treatment duration, the need for additional interventions, and overall healthcare resource utilization.

Overall, the findings of this review corroborate the role of NPWT as a promising, safe, and potentially superior intervention compared with traditional approaches in specific contexts of partial- and full-thickness burns. However, the heterogeneity of the study designs, populations evaluated, and therapeutic protocols underscores the need for additional randomized controlled trials with greater methodological rigor and standardized outcomes to strengthen the level of evidence and broaden the clinical applicability of these results.

Observational studies without control groups^21,22^ were included for descriptive purposes, but they did not allow for direct inference regarding comparative effectiveness. The reported outcomes indicated successful graft take and favorable scar quality.

## Conclusion

The systematic review demonstrated that NPWT is associated with favorable outcomes in the treatment of partial- and full-thickness burns, including reduction in wound area, decreased bacterial load, and accelerated re-epithelialization time when compared with conventional approaches. In addition, NPWT showed a positive impact on skin graft integration and overall improvement in healing quality, particularly when applied at strategic stages of surgical and reconstructive management. Taken together, these findings suggest that NPWT may serve as an adjunctive strategy in selected burn scenarios, particularly graft stabilization, and infection control are clinical priorities.

## Data Availability

All datasets were generated or analyzed in the current study.
